# Interpretation of PET/CT findings in patients with advanced lung cancer who have undergone pleurodesis

**DOI:** 10.3332/ecancer.2014.452

**Published:** 2014-08-12

**Authors:** Marcus Paulo Fernandes Amarante, Riad Naim Younes, Letícia Rigo, Marcelo Rocha de Sousa Cruz

**Affiliations:** 1 Clinical Oncology Department, Antônio Ermírio de Moraes Cancer Centre, Beneficência Portuguesa, São Paulo 01321-001, Brazil; 2 Medimagem, Beneficência Portuguesa, São Paulo 01321-001, Brazil

**Keywords:** PET/CT, lung cancer, pleurodesis

## Abstract

A consensus has not yet been reached for the ideal moment to carry out pleurodesis in patients with malignant pleural effusion among the majority of centres, especially those which don’t specialise in oncologic treatment. The PET (positron emission tomography)/CT (computed tomography) used in the staging of patients with non-small-cell lung cancer (NSCLC) and small-cell lung cancer (SCLC) has caused controversy when used in the evaluation of therapeutical response and in detection of recurrence in patients with pleurodesis. For not distinguishing between inflammatory and neoplasic processes while using PET or CT, suspicion of pleural involvement can result in the indication for invasive diagnostic procedures or inadequate exchange of therapy. In such cases, the hypothesis of the inflammatory process must be included in the differential diagnoses for positive findings with the PET/CT in patients with NSCLC who have undergone pleurodesis, independently of time since the procedure. The reports of two patients with NSCLC have been presented in order to illustrate situations in which pleurodesis has been performed at the moment of diagnosis, outside of a cancer centre.

## Introduction

Pleurodesis is considered to be a safe and effective form of treatment in patients with pleural effusions (PEs), commonly observed in patients with thoracic malignancies [1–3]. A consensus has not yet been reached among the majority of centres regarding the ideal time for carrying out this procedure, especially among those who do not specialise in oncologic treatment.

The prognosis of patients with lung cancer is related to the stage of the disease at diagnosis. The PET/CT, a fusion of tomography images by positron emission with 18F fluorodeoxyglucose (FDG-PET) and computed tomography (CT), is indicated for the staging of patients with non-small-cell lung cancer (NSCLC) and small-cell lung cancer (SCLC) [4–6]. However, its role in the assessment of therapeutic response and detection of recurrence in patients with a history of pleurodesis remains controversial.

The PET/CT may be unable to distinguish between inflammatory processes resulting from prior pleurodesis and neoplastic processes. Thus, suspicion of pleural involvement or pleural disease progression may result in the indication of invasive diagnostic investigation or even inadequate exchange of therapy [7].

To illustrate these situations, we present the following two case reports of patients diagnosed with NSCLC and pleurodesis performed upon diagnosis outside the reference centre for the treatment of cancer.

## Presentation of cases

### Patient 1

The first patient was a 38-year-old male, non-smoker, with a history of pain in his left hemithorax. He presented at the emergency room of a general hospital because of sudden chest pain. The x-ray revealed PE on the left side, affecting one-third of the hemithorax. He underwent thoracentesis with thoracoscopy, which showed diffuse pleural involvement. The patient underwent pleurodesis catheterization on the same occasion, but without undergoing a review by a multidisciplinary team specialising in oncology.

Anatomopathological examination revealed an adenocarcinoma of metastatic lung in the pleural fragment. The post-pleurodesis PET/CT performed revealed a nodule in the superior segment of the lower lobe of the left lung and prominent lymph nodes in the ipsilateral hilum, mediastinum (right inferior and superior, pre-vascular, and subcarinal paratracheal chains), and abdomen (celiac, trunk, and left paraaortic chain), all displaying glycolytic hypermetabolism. A diffuse and irregular pleural thickening of the left hemithorax was also observed, predominantly at the base, revealing radiopharmaceutical uptake with standardised uptake value (SUV) 12.7. There was no evidence of metastases in other organs (Figure 1a).

The epidermal growth factor receptor (EGFR) evaluation showed a deletion in exon 19, and the patient received first-line therapy with the tyrosine kinase inhibitor of the EGFR erlotinib. The patient had a grade 2 skin rash three days after the start of medication, which remained throughout the duration of the treatment.

After four months of treatment, the patient was clinically well, with no new complaints. A new PET/CT was carried out, and it identified the permanence of the diffuse and nodular pleural thickening, predominantly at the base, but showing increased radiopharmaceutical uptake (SUV 17.7) (Figure 1b).

### Patient 2

The second patient was a 65-year-old female, non-smoker, with a history of fatigue and dyspnea for 20 days prior to presentation. She presented at the emergency room of a general hospital, where a medical investigation was performed and showed evidence of left PE. She underwent thoracentesis, which also showed signs of diffuse pleural involvement. The patient then underwent pleurodesis by the video-assisted thoracoscopic surgery (VATS) technique. A pleural biopsy showed a poorly differentiated adenocarcinoma of the lung.

The initial PET/CT, performed after the pleurodesis, showed multiple nodules in both lung fields, measuring up to 0.6 cm, with no significant FDG uptake, presumably due to its small dimensions. There were also mediastinal lymph nodes, some showing radiotracer uptake, and a left retropectoral lymph node showing moderate avidity to glucose, as well as diffuse pleural thickening on the left, with nodulariformes components, and pronounced glycolytic metabolism (SUV 11.2). Additionally, bone metastases were observed in the lumbar and left iliac region.

The EGFR evaluation showed deletion in exon 19, and the patient received therapy with erlotinib. A clinical reassessment was performed every 20–30 days with chest CT every two months, showing objective response to treatment. After nine months, due to complaints of chest pain on the left side, we chose to perform a new PET/CT. Despite the findings that confirmed disease progression with the emergence of new pulmonary nodules, there was a moderate reduction of the diffuse pleural thickening on the left, but with increased FDG uptake (SUV 13.3) (Figure 2a).

The use of erlotinib was suspended, and chemotherapy was initiated with a regimen of carboplatin, paclitaxel, and bevacizumab. After six cycles of this treatment regimen, despite the partial response according to the response evaluation criteria in solid tumours (RECIST), the PET/CT showed persistent signs of pleurodesis in the base of the left hemithorax, keeping the radiopharmaceutical uptake as SUV 13.1 (Figure 2b).

## Discussion

In lung cancer, incidence of malignant PE at diagnosis is 150 to 175,000 cases per year in the United States. Malignant effusions occur in 7–15% of all patients with lung cancer [8]. A new classification of the International Association for the Study of Lung Cancer (IASLC) classifies malignant PE as stage IV [9].

The recommendation for management of malignant PE is to proceed with thoracentesis at diagnosis for pathological confirmation of malignancy. Pleurodesis is indicated for patients with recurrent PE, dyspnea, and who do not respond to basic treatment of neoplasia [10, 11].

Talc is the most common material used for this procedure. It is hydrous magnesium silicate and causes an intrapleural inflammatory response capable of causing adhesions [12]. Pleural talc deposits are often described in CT as areas of increased attenuation, most commonly observed in the basal and posterior regions of the pleural space [13].

The main access routes for pleurodesis are VATS and chest drainage with small calibre catheter by thoracic puncture. The first is at least as effective, and in some studies, significantly more effective than the latter [14].

Since the first description by Murray *et al*, several authors described the appearance of FDG-PET after pleurodesis [15, 16]. During follow-up, although changes in pleural thickening by computed tomography tend to stabilise at five months after pleurodesis, SUV values seemed to persist and increase even further for several years [17]. In the literature, the increased pleural uptake by FDG-PET is described for more than ten years [18], for up to 48 years after the procedure [19].

In the reported cases, the time of pleurodesis appears to have been inappropriate and could have generated a confounding factor in the objective assessment of treatment response for cases with predominant pleural metastases. However, it is noteworthy that there are other parameters that could be used in these cases to assess the response, such as the size of the pulmonary nodules, and especially the size and SUV of mediastinal lymph nodes.

RECIST, in its 2009 edition (RECIST 1.1), does not consider pleural disease in this review, in which these cases also suffered interference from the inflammatory process and thickening caused by pleurodesis [20]. On the other hand, it is noteworthy that the majority of pleural metastases are smaller than 1 cm each, which is not well assessed by PET-CT [21]. A false-positive staging can prevent proper management of the patient.

## Conclusion

The close clinical correlation of PET/CT and chest CT since the beginning of the diagnostic investigation in NSCLC is critical to the successful treatment of the patient. Pleurodesis is not indicated at diagnosis, but only in MPD recurrence, or for those patients who do not respond to basic treatment. Once pleurodesis is performed, the hypothesis of an inflammatory process should always be included in the differential diagnoses for positive findings in PET/CT of patients with NSCLC, regardless of the elapsed time of the procedure. Furthermore, even in patients who presented with predominant pleural disease at diagnosis, pleural SUV should not be used as a parameter for assessing response to treatment.

## Figures and Tables

**Figure 1a.**
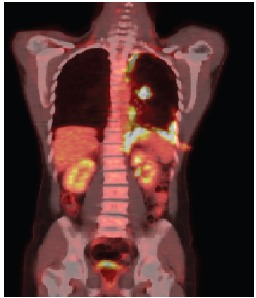
PET–CT demonstrating the absorption in the left lung nodule and pleural thickening of the left base.

**Figure 1b.**
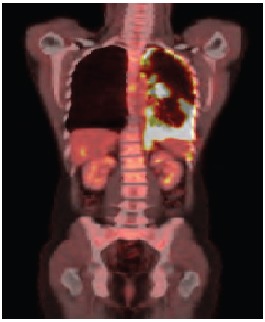
PET–CT showing evidence of the presence of pleural thickening and increase of radiopharmaceutical capture.

**Figure 2a.**
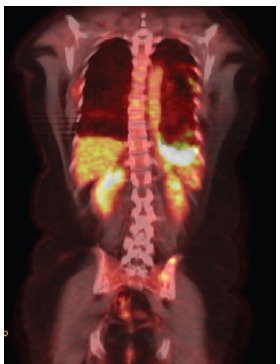
PET–CT after erlotinibe with reduction of thickening in the left pleural and increase on the FDG capture.

**Figure 2b.**
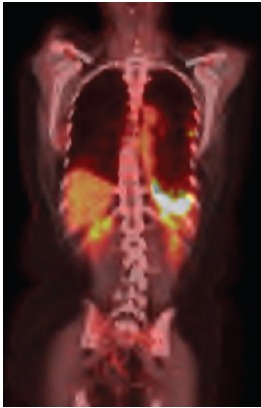
PET–CT after second–line therapy revealing radiopharmaceutical captation maintanence.
